# Effectiveness of risk minimisation measures for valproate: A cross‐sectional survey among physicians in Europe

**DOI:** 10.1002/pds.5119

**Published:** 2020-11-20

**Authors:** Massoud Toussi, Bardoulat Isabelle, Stephanie Tcherny‐Lessenot, Hanka de Voogd, Vasilis Dimos, Sigal Kaplan

**Affiliations:** ^1^ Real World Evidence Solutions IQVIA La Défense Cedex France; ^2^ Epidemiology and Benefit‐Risk Sanofi Aventis R&D Chilly‐Mazarin France; ^3^ Global Clinical Research Mylan Amstelveen, The Netherlands; ^4^ Department of Pharmacovigilance (medical section) Demo S.A. Athens Greece; ^5^ Global Patient Safety & Pharmacovigilance Teva Pharmaceutical Industries Ltd Netanya Israel

**Keywords:** Direct Healthcare Professional Communication, pharmacoepidemiology, prescribing behaviour, risk minimisation measures, valproate

## Abstract

**Purpose:**

This study evaluated the effectiveness of risk minimisation measures (RMMs) implemented following the 2014 referral for valproate in Europe.

**Methods:**

Cross‐sectional survey was conducted over 2‐month period in 2016 among physicians who prescribed valproate in France, Germany, the United Kingdom, Spain and Sweden. The web‐based questionnaire included five endpoints to evaluate physicians' knowledge on (a) prescribing valproate only for epilepsy and bipolar disorder in women if other treatments were ineffective or not tolerated; (b) ensuring supervision by experienced physicians while treating these conditions; (c) considering alternative treatments for women planning pregnancy, regular review of treatment needs and re‐assessing the benefit–risk balance in women and girls reaching puberty; (d) informing patients about the risks of taking valproate during pregnancy and (e) advising women on effective contraception during their treatment.

**Results:**

Among 1153 physicians, 95.5% responded prescribing valproate for epilepsy and bipolar disorder in women only if other treatments are ineffective/not tolerated; 66.5% supervised while treatment; 76.6% considered alternative treatments for women planning pregnancy; 92.1% informed patients about the risks of taking valproate during pregnancy and 94.4% advised patients on the use of effective contraception during its treatment. Overall, 25.8% physicians recalled receiving both educational material (EM) and Dear Healthcare Professional Communication (DHPC). All endpoint rates were higher for physicians who acknowledged receipt of both DHPC and EM compared to physicians who did not receive them.

**Conclusions:**

Although results varied across geography and physician speciality, majority of physicians had good knowledge about the indication and safety aspects of prescribing and using valproate.

**KEY POINTS**

- Majority of physicians had good knowledge about prescribing valproate for epilepsy and bipolar disorder in women of child‐bearing potential (WCBP).
- Physicians acknowledging receipt of RMMs materials had better knowledge regarding appropriate prescribing of valproate compared to physicians who did not acknowledge receipt.
- Overall, majority of physicians correctly responded to the five key primary endpoints ranging from 66.5% to 95.5%.

## 1 INTRODUCTION

Valproate and related substances (ie, sodium valproate, valproic acid, magnesium valproate, valproate, valpromide and valproate semi sodium) were first licensed in Europe for the indication of epilepsy and bipolar disorder in the 1960s. Valproate is also indicated for prophylaxis of migraine attack in few European countries. However, following approval of valproate, concerns were raised about the risk of teratogenicity associated with valproate use during pregnancy.^1^ A systematic review and meta‐analysis in pregnant women receiving antiepileptics drugs (AEDs) for epilepsy showed valproate monotherapy to be associated with the highest incidence of congenital malformations (10.7%), followed by phenytoin (7.3%).^2^ Also, children born to women who were exposed to valproate and/or related substance during pregnancy were at risk of serious developmental disorders related to language, intellectual and memory abilities.^2^, ^3^, ^4^, ^5^

Considering the evidence associated with high risk of congenital malformations and neurodevelopmental disorders related to valproate and/or related substances exposure in utero, the European Medicines Agency (EMA) in November 2014 put forth recommendations strengthening the warnings on use of valproate in women of child‐bearing potential (WCBP), pregnant women and female children to ensure benefits outweigh the risk of valproate and/or related products.^6^ Risk minimisation measures (RMMs) included educational material (EM) and Direct Healthcare Professional Communication' (DHPC). These included prescriber guide, patient booklet and information ensuring the understanding and the awareness of prescribers and patients on the risks of valproate exposure during the pregnancy.^7^ The RMMs were implemented to communicate and highlight specific restrictions regarding the use of valproate and related substances as follows^8^: (a) Valproate should not be used to treat epilepsy or bipolar disorder in girls and in women who are pregnant or who can become pregnant, unless other treatments are ineffective or not tolerated; (b) women for whom valproate is the only option after trying other treatments should use effective contraception; (c) treatment should be started and supervised by a doctor experienced in treating these conditions; (d) women who have been prescribed valproate should not stop taking their medicine without consulting their doctor first; (e) in countries where medicines constituting valproate are authorised for the prevention of migraine, women must not use valproate for preventing migraine when they are pregnant.^8^ Pregnancy should be excluded before starting treatment for migraine, and women should use effective contraception; (f) physicians who prescribe valproate should provide women with complete information enabling them to fully understand of the risks of the treatment regarding pregnancy.^8^

As part of the risk management plan for valproate, a post‐authorisation safety study (PASS) survey was conducted among healthcare professionals (HCPs), in parallel to a real‐world drug utilisation study (DUS). The objective of this PASS survey was to evaluate the effectiveness of the RMMs and specifically to evaluate the knowledge and behaviour of HCP regarding the latest prescribing conditions and safety information about valproate and related substances.

## 2 METHODS

### 2.1 Study design

This was a multinational, cross‐sectional, web‐based survey (European Union electronic Register of Post‐Authorisation Studies, EU‐PAS Register: EUPAS11379) conducted among physicians from various specialities (GPs, neurologists, psychiatrists, internists/paediatricians) who prescribed valproate within the 12 months prior to the survey. The survey was conducted in five European countries, France, Germany, Spain, Sweden and the United Kingdom (UK), between 17th June 2016 and 25th August 2016.

We aimed to recruit a total of 1387 physicians (324 each from France, Germany and the UK, 260 from Spain and 155 from Sweden), in order to assess at least 1067 analysable questionnaires, assuming a 30% rate of non‐analysable questionnaires. The sample size was calculated to achieve a minimum of 3% precision level for a proportion of 50% within 95% CI. Invitations to complete the survey were sent via e‐mail to a randomly selected sample of physicians. Physicians were identified according to their speciality from a comprehensive list in the IQVIA OneKey database, which is a global reference healthcare professional database providing comprehensive and integrated information of more than 15 million HCPs across 100 countries. OneKey continuously updates the accuracy and level of details in the database through a dedicated team of 700 experts.^9^ The physician selection was random, and no pre‐registration was required by the physicians. Furthermore, there was no involvement of any pharmaceutical company. Physicians accessed the survey in the study website through a distinct identification number and password. All physicians who participated in the web‐based survey were requested to complete an informed consent form. A screening log was maintained to follow‐up on the inclusion of physicians in the survey over time. Physicians were offered a fair market value compensation (which they could opt out voluntarily) in return for the time spent participating in this survey according to the European Pharmaceutical Marketing Research Association (EphMRA) recommendations and the Association of Opinion and Behaviour in health field research companies (ASOCS) charter. To ensure good response rate, the targeted physicians were called and sent e‐mail reminders through experienced operators before they were considered not reachable.

### 2.2 Survey methodology

A web‐based anonymous survey questionnaire was developed and consisted of 21 close‐ended multiple‐choice questions, including eligibility questions, questions on physicians' demographics and practice information, questions on the receipt of the DHPC and EM and questions on physicians' knowledge of the latest primary prescribing conditions and safety information about valproate as presented in the DHPC and EM (Supporting Information). Physicians were also enquired about information related to up to five consecutives prescriptions of valproate to female patients. The physicians were asked to recall prescription cases from memory to keep the study pragmatically simple and within the definitions of a survey. The extent of knowledge of surveyed physicians for measures included in the RMMs was based on five primary endpoints (Table 1). The levels of knowledge, receipt, use and implementation were calculated as the proportions of physicians responding “yes” to questions pertinent to the prescribing conditions and safe use of valproate and related substances.

**TABLE 1:** Main primary endpoints in the survey

Primary endpoints	Definition
01	Understanding on prescribing valproate for epilepsy and bipolar disorder in women if other treatments are ineffective or not tolerated.
02	Ensuring that a doctor experienced in treating these conditions supervises the treatment of epilepsy or bipolar disorder.
03	Taking into consideration alternative treatments if a woman becomes or plans to become pregnant during valproate treatment, regularly review the need for treatment and re‐assess the balance of the benefits and risks for women and also for girls reaching puberty who are taking valproate.
04	Informing patients of the risks of taking valproate during pregnancy.
05	Advising women taking valproate medicines about effective contraception during their treatment.

The questionnaire was provided in English and local language in each participating country. The survey was translated to local languages using forth and back method, that is, a person translated it from English to the destination language and then another person translated back the questionnaire to English. It was then compared for inconsistencies with the original English version. The questionnaire was pre‐tested for its consistency, comprehensibility and appropriateness of medical terms among six physicians before its implementation. To minimise the opportunity for physicians to search for answers or to change their replies upon the content in successive questions, physicians could not revise/change the answers to preceding questions. The time for its completion was up to 20 minutes.

### 2.3 Statistical methods

All analyses were descriptive in nature. The primary analysis included the population set of all physicians who met the eligibility criteria and answered all questions in the questionnaire. No statistical comparison were made, since no hypothesis testing was performed in the current study. Categorical variables were described as the total number and relative percentage per category. Quantitative variables were described with standard statistics including mean and SD. Missing data were displayed when present (value > 0). A random stratified sampling method was applied within stratification by country and speciality. For generalisability, the survey results were weighted to project the proportion (or results) to the entire population within the country and by speciality. A weight variable was applied to the analysable units during the calculation of result, to correct any over‐ or under‐sampling that may have occurred for a country or speciality. Analyses were performed overall, by speciality and by country. Data analyses were conducted using the SAS software V9.4 (SAS Institute, Inc., Cary, North Carolina).

## 3 RESULTS

### 3.1 Demographics and clinical practice characteristics

Of 1526 physicians who agreed to participate in the survey, a total of 1153 physicians submitted a complete and analysable questionnaire, of them 255 (22.1%) were from France, 254 (22.0%) from Germany, 244 (21.1%) from Spain, 136 (11.7%) from Sweden and 264 (22.8%) were from the UK. The overall response rate among physicians was highest in Spain (99.7%) and lowest in France (20.2%) (Table 2).

**TABLE 2:** Physician attrition details

Parameters	France	Germany	Spain	Sweden	UK
Physicians targeted	12 389	8796	4256	1215	8059
Physicians not reachable	10 800	7521	3949	1042	7536
Physicians contacted	1589	1275	307	173	523
Physicians who refused to participate	1268	936	1	3	133
Physicians who agreed to participate	321	339	306	170	390
Physicians excluded	66	85	62	34	126
1. Failed screening (the doctor does not meet the criteria as set by the specific project)	40	50	28	27	69
2. Survey initiated but the physician does not complete the whole survey on his own initiative	26	35	34	7	57
Physicians with complete questionnaire	255	254	244	136	264
Contact rate	12.8%	14.5%	7.2%	14.24%	6.5%
Response rate	20.2%	26.6%	99.7%	98.3%	74.6%
Refusal rate	10.2%	10.6%	0.02%	0.2%	1.7%

*Note*: Contact rate = contacted physicians/targeted physicians; response rate = physicians who agreed to participate/contacted physicians; refusal rate = (contacted physicians who agreed to participate)/physicians targeted; targeted physicians: physicians to whom an e‐mail has been sent or phone call was made; contacted physicians: physicians who have been reached out by phone or have opened their e‐mail (if the score is technically available in their country); physicians who agreed to participate: physicians willing to participate in the survey (eg, by phone or by clicking on the link provided in the recruitment e‐mail); physicians with complete questionnaire: physicians who actually completed the questionnaire until its end.

Overall, majority of the surveyed physicians with complete and analysable questionnaire were in the 40 to 59‐year age group (70.6%) and had more than 10 years of experience (88.5%). Overall, GPs or family physicians (34.3%) and hospital‐based (46.0%) constituted the largest percentage among all specialities and settings. The mean (±SD) number of valproate prescriptions to female patients per physician prescribed within the previous year was 48.2 (±100.5) (Table 3).

**TABLE 3:** Surveyed physician's demographics and practice characteristics (unweighted)

	GP (N = 396) (34.3%)	Neurologists (N = 228) (19.7%)	Psychiatrists (N = 233) (20.2%)	Other Specialists (N = 296) (25.6%)	Overall (N = 1153) (100%)
Age					
≤30 years old	2 (0.5%)	6 (2.6%)	4 (1.7%)	2 (0.7%)	14 (1.2%)
31‐39 years old	52 (13.1%)	45 (19.7%)	43 (18.5%)	34 (11.5%)	174 (15.1%)
40‐49 years old	97 (24.5%)	83 (36.4%)	75 (32.2%)	114 (38.5%)	369 (32.0%)
50‐59 years old	180 (45.5%)	73 (32.0%)	78 (33.5%)	114 (38.5%)	445 (38.6%)
≥60 years old	65 (16.4%)	21 (9.2%)	33 (14.2%)	32 (10.8%)	151 (13.1%)
Years in medicine practice					
≤1 year	0 (0.0%)	1 (0.4%)	0 (0.0%)	0 (0.0%)	1 (0.1%)
1‐5 years	13 (3.3%)	5 (2.2%)	8 (3.4%)	3 (1.0%)	29 (2.5%)
6‐10 years	25 (6.3%)	29 (12.7%)	26 (11.2%)	23 (7.8%)	103 (8.9%)
>10 years	358 (90.4%)	193 (84.6%)	199 (85.4%)	270 (91.2%)	1020 (88.5%)
Setting					
Office based	280 (70.7%)	41 (18.0%)	51 (21.9%)	47 (15.9%)	419 (36.3%)
Hospital based	81 (20.5%)	140 (61.4%)	114 (48.9%)	195 (65.9%)	530 (46.0%)
Both office and hospital based	35 (8.8%)	47 (20.6%)	68 (29.2%)	54 (18.2%)	204 (17.7%)
Number of prescriptions to female patients within the last year					
Mean (±SD)	36.9 (71.7)	65.9 (118.5)	66.5 (137.0)	35.4 (78.4)	48.2 (100.5)

Abbreviation: GP, general practitioner.

In the UK, the highest proportion of physicians were in the age group of 40 to 49 years. In all countries, except Spain, GPs were mainly office‐based.

### 3.2 Knowledge of the prescribing conditions and safety information warnings of valproate

A total of 86.5%, 75.9%, and 62.5% physicians responded to valproate prescribing indication to be epilepsy with generalised seizures, epilepsy with partial seizures with and without secondary generalisation and bipolar disorders, respectively. In all the participating countries, neurologists were found to be best‐informed speciality (overall 96.1%) for epilepsy with generalised seizures indication. However, psychiatrists were the best‐informed for the bipolar disorders' indication (99.1%). (Table S1).

**FIGURE 1:**
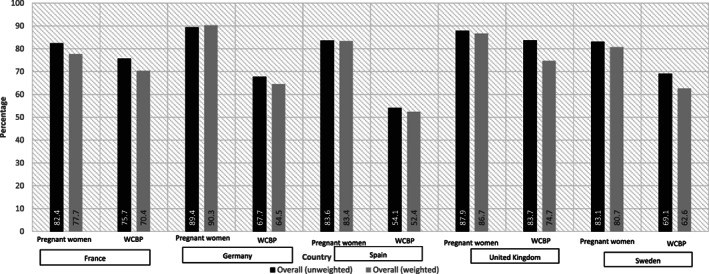
Knowledge that valproate should not be prescribed to pregnant women or women of child‐bearing potential unless other treatments are ineffective or not tolerated. WCBP, women of child‐bearing potential

Overall, 84.5% physicians correctly responded that valproate should not be prescribed to pregnant women unless other treatments are ineffective or not tolerated, with the highest proportions in Germany (89.4%) and the UK (87.9%). Considering WCBP, population highest proportion of correct response was obtained in the UK (83.7%) and France (75.7%) (Figure 1). Among specialists, neurologists were less knowledgeable of this condition (80.0%‐87.5%) (Figure S1).

**FIGURE 2:**
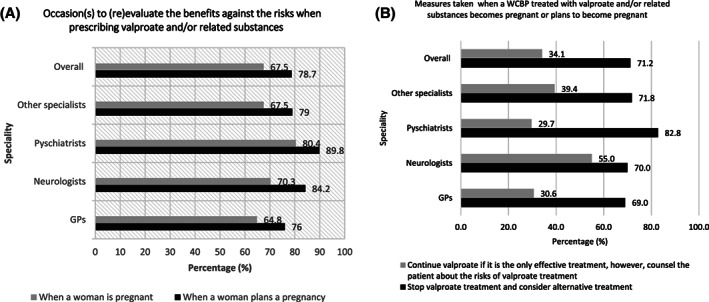
Benefits vs risk for valproate/related substances. GP, general practitioner; WCBP, women of child‐bearing potential

Among all specialities, 78.7% of the physicians weighed the benefits against risks while prescribing valproate and/or related substances when a woman planned her pregnancy. Across all specialities, the weighing of benefit against risk assessment when a woman planned her pregnancy was highest among psychiatrists (89.8%) and least among GPs (76%) (Figure 2A).

Overall, 71.2% of the physicians responded stopping valproate treatment and considering alternative treatment if a woman treated with valproate and/or related substances becomes pregnant or plans to become pregnant. However, 34.1% of the HCPs in all surveyed countries continued valproate if it is the only effective treatment, however, counselled the patient about its risks. Among all the specialities, highest response for stopping valproate was found in psychiatrists (82.8%) and least for GPs (69.0%) (Figure 2B).

### 3.3 Endpoints related to behaviour

**FIGURE 3:**
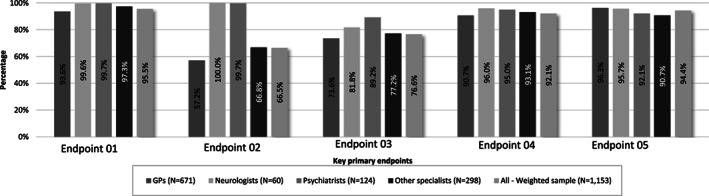
Analysis of success with results on the five key primary endpoints separately for the overall weighted sample according to physician's speciality. GP, general practitioner; endpoint 01: Understanding on prescribing valproate for epilepsy and bipolar disorder in women if other treatments are ineffective or not tolerated; endpoint 02: Ensuring that a doctor experienced in treating these conditions supervises the treatment of epilepsy or bipolar disorder; endpoint 03: Taking into consideration alternative treatments if a woman becomes or plans to become pregnant during valproate treatment, regularly review the need for treatment and re‐assess the balance of the benefits and risks for women and also for girls reaching puberty who are taking valproate; endpoint 04: Informing patients of the risks of taking valproate during pregnancy; endpoint 05: Advising women taking valproate medicines about effective contraception during their treatment

**FIGURE 4:**
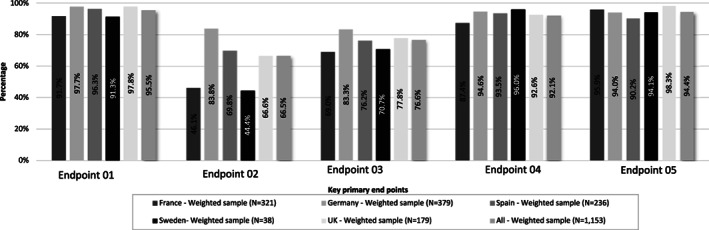
Analysis of success with results on the five key primary endpoints separately for the overall weighted sample according to country. Endpoint 01: Understanding on prescribing valproate for epilepsy and bipolar disorder in women if other treatments are ineffective or not tolerated; endpoint 02: Ensuring that a doctor experienced in treating these conditions supervises the treatment of epilepsy or bipolar disorder; endpoint 03: Taking into consideration alternative treatments if a woman becomes or plans to become pregnant during valproate treatment, regularly review the need for treatment and re‐assess the balance of the benefits and risks for women and also for girls reaching puberty who are taking valproate; endpoint 04: Informing patients of the risks of taking valproate during pregnancy; endpoint 05: Advising women taking valproate medicines about effective contraception during their treatment

**FIGURE 5:**
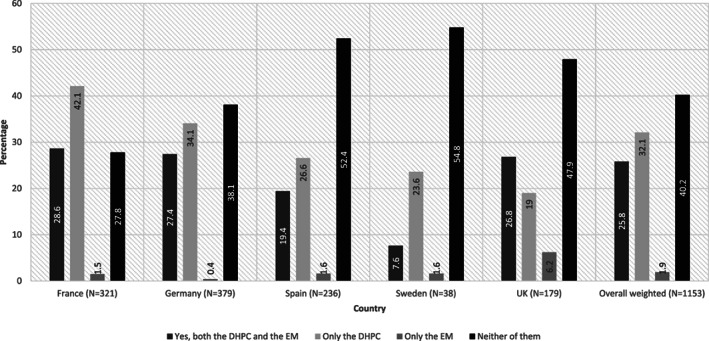
Acknowledgement of receipt of DHPC and/or EM related to valproate. DHPC, Dear Healthcare Professionals Communication; EM, educational material

A total of 95.5% respondents prescribed valproate for epilepsy and bipolar disorder in women if other treatments were ineffective or not tolerated. Overall, majority of respondents (95.5%) fulfilled the first primary endpoint. The proportion of physicians responding to lowest for second and third endpoint, that is, in 66.5% and 76.6% physicians, respectively. Among all the specialities, GPs responded the least for endpoint 2 (57.2%) and 3 (73.6%) (Figure 3). Among all the countries, France had the least response rate for second (46.1%) and third endpoint (69.0%) (Figure 5). A total of 92.1% of the physicians always informed patients about the risks of taking the drug during pregnancy before prescribing valproate and/or related substances to a woman of child‐bearing potential (fourth endpoint), and 94.4% advised their patients on the use of effective contraception during valproate treatment (fifth endpoint) (Figure 4).

### 3.4 Acknowledgement of receipt of DHPC and EM

Overall, 25.8% of physicians acknowledged receipt of both DHPC and EM related to valproate and/or related substances. Physicians more commonly reported receipt of DHPC (57.9%) than EM (27.7%). Overall, 40.2% of physicians did not recall having received either the DHPC or the EM with a highest proportion of such physicians in Sweden (54.8%) and least in France (27.8%) (Figure 5). The highest percentage of physician specialities acknowledging receiving both DHPC and EM were neurologists (50.7%), followed by psychiatrists (43.8%) (Table S2).

Physicians who acknowledged receipt of both DHPC and EM had higher rates of correct answers to key endpoint questions (78.9%‐97.6%) than those who mentioned they had not received them (60.4%‐93.2%).

## 4 DISCUSSION

This is the first survey study assessing the effectiveness of RMMs for valproate and related substances. The RMMs were developed and implemented to ensure correct knowledge of physicians about prescribing conditions and safe use when considering prescribing valproate in European countries. The study showed majority of physicians (86.5%) responding that valproate should be prescribed for epilepsy with generalised seizures. Over 90% of respondents (a) prescribed valproate for epilepsy and bipolar disorder in women if other treatments were ineffective or not tolerated; (b) informed patients of the risks of taking valproate during pregnancy and (c) advised women taking valproate medicines about effective contraception during their treatment. Also, a large proportion of physicians considered alternative treatments in case a woman becomes or plans to become pregnant during valproate treatment, regularly reviewed the need for treatment and re‐assessed the balance of the benefits and risks for women and also for girls reaching puberty who are taking valproate. About one third of the respondents, mainly GPs and other specialties, did not ensure that a doctor experienced in treating these conditions supervised the treatment of epilepsy or bipolar disorder. Furthermore, only one third of respondents continued valproate if it was the only effective treatment and counselled the patients about its risks, while the majority of physicians (71.2%) responded stopping valproate treatment and considered alternative treatment.

Among all the countries, the primary endpoint of advising women taking valproate medicines about effective contraception during their treatment was more commonly met compared to the other endpoints for France and the UK (ie, 95.9% and 98.3%, respectively). Though the overall findings suggest that physicians were knowledgeable, there are still areas where knowledge among physicians about pregnancy in female patients treated with valproate could be improved.

More than 75% of surveyed physicians who acknowledged receiving both DHPC and the EM or only the DHPC or EM have fulfilled the primary endpoints of the study when compared to those who mentioned they had neither received the DHPC nor EM. Individual primary endpoints defined for the study had better results for physicians who have acknowledged receipt DHPC and/or EM compared to those who did not, suggesting the effectiveness of RMMs when read. Variations in results of acknowledging the receipt of RMMs material indicated differences in RMMs material distribution among the countries.

Risk minimisation measures are an integral part of the EMA pharmacovigilance legislation.^10^ Once RMMs are in place, the Marketing Authorisation Holders (MAHs) are often required to monitor the effectiveness of RMMs, as a condition of market authorisation.^10^ It is not only a crucial aspect of the continuous pharmacovigilance regulatory framework but also provides useful insight into HCP's knowledge and compliance towards safety communication materials for the approved product in EU region.^11^ Surveys studies are a robust and versatile way to evaluate RMMs and can also be used to measure self‐reported behaviour.^12^ Previously published European region surveys for various approved products have helped in identification of physician speciality and/or region in which the RMMs should be strengthened or intensified, in addition to assessing the HCPs knowledge.^13^, ^14^, ^15^, ^16^ Knowledge from these studies can help the national health authorities to facilitate the improvement of safety communication strategies.^16^

The current study had several strengths. The survey questionnaire was tested for clarity, consistency comprehensibility and appropriateness of medical terms before the data collection. The enrolment from each country provided the participation from various specialities, thereby providing a generalisable view for this secure web‐based survey. The access to the questionnaire was limited to physicians who were invited to participate in the survey through the link they received by e‐mail. The link was only activated if clicked from the e‐mail. Thus, assessment of selection bias or unverified respondent inclusions were not applicable for the current study.

The average response rate in this study was high but varied considerably from 20.2% (France) to 99.7% (Spain). The highest percentage of respondent physicians was GPs followed by other specialities (eg, neurologists). Compared to other survey studies measuring the effectiveness of RMMs in the European region, our study showed comparatively higher response rate.^17^, ^18^, ^19^ Though there was an intentional over‐sampling in Sweden and Spain, and of less frequent specialities as neurologists, psychiatrists and an under‐sampling in Germany, France and of GPs, the sample data were not adequate for generalisability to the overall target population. Hence, the results of the study sample were adjusted for sampling approach and were weighted, to allow for generalisability of the study results to the target population.

Few limitations inherent in cross‐sectional surveys, that is, social desirability and selection bias cannot be ruled out. A web‐based survey tends to foster social desirability bias, that is, tendency of physicians to give socially desirable responses instead of reflecting their understanding or knowledge. Social desirability impacts the validity of survey measures, thus inducing misleading results. However, the use of pre‐populated structured items in the questionnaire tends to reduce this bias.^20^, ^21^ The potential for selection bias due to voluntary participation of targeted physicians is another inherent bias in web‐based surveys which affects the quality of the survey and leads to unreliable results and inferences.^22^, ^23^ Hence, to quantify any selection bias, the distribution of stratification criterion of physicians was compared between participants and non‐participants. Furthermore, in order to reach the target per physician speciality and country, efforts were made to contact the required specialists.

This survey reflected physician's knowledge and perception about treatment rather than the actual prescribing pattern of valproate in WCBP. Therefore, a DUS was parallelly conducted to confirm the actual prescribing patterns among WCBP. The findings of both studies, other utilisation studies and clinical insights led to 2018 EMA requirements to conduct additional DUS and survey to characterise the nature and the extent of the risks by valproate and/or related substances, following the strengthening of RMMs.^24^

In summary, the study revealed that despite geographical and speciality‐based variations for the knowledge of indication and safety aspects of valproate, majority of the surveyed physicians appropriately understood the prescribing indications of valproate and related substances in relation to WCBP and pregnant women.

## ETHICAL STATEMENT

The survey followed the regulatory and ethical requirements of each country. The survey complied with the module VIII of the good pharmacovigilance practices (GVP). The study followed the European Pharmaceutical Marketing Research Association (EphMRA) code of conduct guidelines for all countries. Due to the survey nature of this study, ethical review was not required. Additionally, no legal approvals or information was required for the five countries.

## CONFLICT OF INTEREST

M. Toussi, I. Bardoulat are salaried employees of IQVIA (La Défense, France) a human data science company which received funds for the conduct of the study. Stephanie Tcherny‐Lessenot is an employee of Sanofi Aventis R&D (France). Sigalit Kaplan is an employee of Teva Pharmaceutical Industries Ltd. (Israel). Hanka de Voogd is an employee of Mylan EPD (The Netherlands). Vasilis Dimos is an employee of DEMO S.A. (Greece).

## Supporting information


**Data S1.** Supporting Information.
