# Supplementary material for: Effectiveness of risk minimisation measures for valproate: A cross‐sectional survey among physicians in Europe
Source: Pharmacoepidemiol Drug Saf. 2020 Nov 20;30(3):283–91. doi: 10.1002/pds.5119 (PMC7894483; doi:10.1002/pds.5119)
Supplement: Supplementary file 1 — Data S1. Supporting Information. [file PDS-30-283-s001.docx]

# SUPPLEMENTARY MATERIAL

**Supplementary material 01**

Evaluation of the Effectiveness of Risk Minimisation Measures: A Joint PASS Survey among Health Care Professionals to Assess their Knowledge and Attitudes on Prescribing Conditions of Valproate in France, Germany, United Kingdom, Spain and Sweden

**Web questionnaire**

**Physician Demographics and practice information**

**D1**. What is your age category?

| ≤ 30 years old | ( ) |
| --- | --- |
| 31-39 years old | ( ) |
| 40-49 years old | ( ) |
| 50-59 years old | ( ) |
| ≥ 60 years old | ( ) |

***Data: single punch***

**D2**. Years in medicine practice

| ≤1 year | ( ) |
| --- | --- |
| 1-5 years | ( ) |
| >5-10 years | ( ) |
| >10 years | ( ) |

***Data: Single punch***

**D3**. What is your main medical speciality?

| General practice (GP)/family physician | ( ) |
| --- | --- |
| Neurology | ( ) |
| Psychiatry | ( ) |
| Internal medicine | ( ) |
| Paediatrics | ( ) |
| Other, please specify…………………… | ( ) |

***Data: single punch***

***Data: list of physicians’ specialties.***

**D3a**. For how long have you been working in this speciality ?

| ≤1 year | ( ) |
| --- | --- |
| 1-5 years | ( ) |
| >5-10 years | ( ) |
| >10 years | ( ) |

***Data: Single punch***

**D4**. In which type of setting do you work the majority of your time?

| Office based | ( ) *Go to Screener* |
| --- | --- |
| Hospital based | ( ) *Go to section D5* |
| Both, office and hospital | ( ) *Go to section D5* |

***Data: single punch***

**D5**. Type of setting (tick all that is applicable).

| Primary care | ( ) |
| --- | --- |
| Secondary care | ( ) |
| Speciality care outpatient | ( ) |
| Hospital/Community care | ( ) |

***Data: Multi punch***

**Screener**

**S1.** Have you prescribed valproate and/or related substances in the last 12 months?

| Yes | ( ) *Continue* |
| --- | --- |
| No | ( ) *End of questionnaire* |

***Data: single punch***

-----------------------------------------------------------------------------------------------------------------------------

***Question for the physicians who have prescribed valproate and/or related substances within the last 12 months***

**SQ1**. Please estimate the number of valproate and/or related substances prescriptions (not patients) that you have written to female patients within the last 12 months.

| …. .. ………. |
| --- |

***Data: Open numeric 0-1000***

**Section 1: Prescribing conditions and safety information/warnings of valproate and related substances**

***Explanatory text***

Please answer the following general questions about the prescribing conditions and safety information of valproate and related substances (valproic acid, sodium valproate, valproate semi sodium, valpromide).

**Q1**. According to your understanding, for which of the following indications/symptoms is valproate and related substances prescribed?

| **Indications** |  |
| --- | --- |
| Epilepsy with generalised seizures | ( ) |
| Epilepsy with partial seizures with and without secondary generalisation | ( ) |
| Bipolar disorders | ( ) |
| Migraine prophylaxis | ( ) |
| Wolfram syndrome | ( ) |
| Other, please specify: …………………………………………………... | ( ) |
| I do not know | ( ) |

***Data: Multi punch
Data: Single punch if ‘I do not know’ is ticked***

***Data: randomise order of indications, always keep ‘other, please specify’ in bottom***

***Data: Repeat Q2 for all ticked indications in Q1***

**Q2**.According to your understanding, in what treatment situations do you perceive valproate and related substances to be prescribed for?

| As: |  |
| --- | --- |
| Initial monotherapy | ( ) |
| Add-on due to inadequate control of first line treatment | ( ) |
| Switch due to intolerance of initial monotherapy treatment | ( ) |
| Switch due to ineffective first line treatment | ( ) |
| Other, please specify: | ( ) |
| I do not know | ( ) |

***Data: Multi punch
Data: Single punch if ‘I do not know’ is ticked***

***Data: randomise order of treatment situations, always keep ‘other, please specify’ in bottom***

The results from the multiple answers to question Q2 successively for bipolar disorders and migraine indications are presented in the Appendix section 15.3 : List of tables (See Tables 15.3 29 and 15.3 31): Section 1: 'Prescribing conditions and safety information/warnings of valproate and related substances' Q2c (Bipolar disorder) and Q2d (Migraine prophylaxis) per country according to physician’s speciality.

**Q3**. According to your understanding, in which situation(s) valproate and related substances **should not** be prescribed to female patients, unless other treatments are ineffective or not tolerated?

*(answer: valproate should not be prescribed to female children, female adolescents, women of childbearing potential or pregnant women unless other treatments are ineffective or not tolerated - S1 DHPC)*

| Female children | ( ) |
| --- | --- |
| Female adolescent | ( ) |
| Female of childbearing potential | ( ) |
| Menopausal female | ( ) |
| Pregnant women | ( ) |
| I do not know | ( ) |

***Data: Multi punch
Data: Single punch if ‘I do not know’ is ticked***

**Q4**. In general, before prescribing valproate and/or related substances to a female of childbearing potential, do you inform her of the risks of taking the drug during pregnancy?

| Yes, always | ( ) |
| --- | --- |
| Yes, from time to time | ( ) |
| No | ( ) |

***Data: Single punch***

**Q5**. In which occasion(s) would you (re)evaluate the benefits against the risks when prescribing valproate and/or related substances

*(answer: Carefully balance the benefits of valproate treatment against the risks when prescribing valproate for the first time, at routine treatment reviews, when a female child reaches puberty and when a woman plans a pregnancy or becomes pregnant)*

| At the initiation | ( ) |
| --- | --- |
| In each routine treatment review | ( ) |
| When a female child reaches puberty | ( ) |
| When a woman plans a pregnancy | ( ) |
| When a woman is pregnant | ( ) |
| When a woman reaches menopause | ( ) |
| I do not know | ( ) |

***Data: Multi punch
Data: Single punch if ‘I do not know’ is ticked***

**Q6**. What would you do if a female patient treated with valproate and/or related substances becomes pregnant or plans to become pregnant?

| Continue valproate treatment | ( ) |
| --- | --- |
| Continue valproate if it is the only the effective treatment, however, counsel the patient about the risks of valproate treatment | ( ) |
| Stop valproate treatment and consider alternative treatment | ( ) |
| I do not know | ( ) |

***Data: Multi punch
Data: Single punch if ‘I do not know’ is ticked***

**Q7a**. Have you received the Dear Healthcare Professional Communication (DHPC) related to valproate and related substances since *[to be adapted to the country, e.g. December 2014 for Germany]*?

| Yes | ( ) |
| --- | --- |
| No | ( ) |

***Data: Single punch***

**Q7b**. Have you received the educational materials (EM) related to valproate and related substances since *[to be adapted to the country, e.g. December 2014 for Germany]*?

| Yes | ( ) |
| --- | --- |
| No | ( ) |

***Data: Single punch***

**Section 2: Information about recent prescriptions**

***Explanatory text***

Please try to recall the last prescriptions of valproate and/or related substances (valproic acid, sodium valproate, valproate semi sodium, valpromide) you wrote to female patients in the last 12 months.

Please begin from the latest prescription and fill-in the details for up to the last **5** prescriptions you have written, possibly without any omission.

Please do not report any prescription written before *e.g. June 2015 (to be adapted to the country).*

Please complete this section with the requested patient information based on your recall. Please be assured that all patient data requested is fully anonymised and will be collated with data provided by other respondents’ and presented to the sponsor in an aggregated and fully anonymised form.

As per the 2011 EphMRA (European Pharmaceutical Market Research Association) code of conduct this information is being collected for this survey purposes only.

***Data: Repeat P1-P11 for 5 patients.***

**P1**. Please provide the approximate date of the prescription

Month (MM) ______ Year (YYYY) ______

□ I do not recall

***Data: Open numeric 2014-2015***

**P2**. Please specify the patient status at the time of prescription:

□ Female children (<13 years)

□ Female of childbearing potential (≥13 and <49 years)

□ Menopausal female (≥49 years)

□ I do not recall

***Data: Single punch***

**P3**. If the patient is a female of childbearing potential, please specify if at the time of prescription:

□ She was pregnant

□ She was planning to be pregnant

□ She was not planning to be pregnant

□ She was using effective methods of contraception

□ She was not using effective methods of contraception

□ I do not recall

***Data: Multiple punch***

***Data: Single punch if ‘I do not recall’ is ticked***

**P4**. Please specify for which indication you prescribed valproate and/or related substances at the time of prescription:

□ Epilepsy

□ Bipolar disorders

□ Migraine prophylaxis

□ Other, please specify........................................................................................................

□ I do not recall

***Data: Single punch***

**P5.** Is this prescription of valproate and/or related substances an initiation or a repeat prescription the day of visit:

□ Initiation: medicine given for the very first time

□ Repeat prescription

□ I do not recall

***Data: Single punch***

**P6.** Treatment situation at the time of prescription:

□ Initial monotherapy

□ Add-on due to inadequate control of previous treatment

□ Switch due to intolerance of previous treatment

□ Switch due to ineffective previous treatment

□ Switch due to poor patient compliance

□ Other, please specify........................................................................................................

□ I do not recall

***Data: Multiple punch***

***Data: Single punch if ‘I do not recall’ is ticked***

**P7.** Main reasons for choosing valproate and/or related substances at the time of prescription:

□ Safety

□ Efficacy

□ Patient not responding to other drugs

□ Patient not tolerating other drugs

□ Other drugs are contraindicated to this patient

□ Better compliance

□ Other reasons, please specify.............................................................................................

□ I do not recall

***Data: Multiple punch***

***Data: Single punch if ‘I do not recall’ is ticked’***

**P8.** If the treatment with valproate and/or related substances has been stopped since the prescription, please specify the reason(s) of discontinuation:

□ Not tolerated

□ Not efficient

□ Poor patient compliance

□ Other reasons, please specify.............................................................................................

□ I do not recall

***Data: Multiple punch***

***Data: Single punch if ‘I do not recall’ is ticked’***

**Supplementary table S1: Knowledge of the prescribing conditions and safety information about valproate presented in the DHPC and EM (weighted)**

|  | **GPs (N=671)** | **Neurologists**  **(N=60)** | **Psychiatrists**  **(N=124)** | **Other specialists (N=298)** | **Overall (N=1153)** |
| --- | --- | --- | --- | --- | --- |
| Epilepsy with generalised seizures | 589.7(87.9%) | 57.7 (96.1%) | 78.3 (63.0%) | 271.6 (91.2%) | 997.3 (86.5%) |
| Epilepsy with partial seizures with and without secondary generalisation | 525.8(78.4%) | 51.3 (85.5%) | 66.1 (53.2%) | 231.3 (77.7%) | 874.6 (75.9%) |
| Bipolar disorders | 429.6 (64.0%) | 42.2 (70.3%) | 123.2 (99.1%) | 125.3 (42.1%) | 720.3 (62.5%) |
| Migraine prophylaxis | 258.0 (38.5%) | 40.4 (67.3%) | 28.7 (23.0%) | 101.8 (34.2%) | 428.9 (37.2%) |

GPs: General Practitioners

Supplementary Figure S1: Knowledge of prescribing restriction (or warning)in pregnant women/women with childbearing potential by speciality and country

**Supplementary table S2: Acknowledgement of receipt of DHPC and/or EM related to valproate**

| **Country** | **Speciality** | **GPs** | **Neurologists** | **Psychiatrists** | **Other specialists** |
| --- | --- | --- | --- | --- | --- |
| **France** |  | **(N=97)** | **(N=56)** | **(N=59)** | **(N=43)** |
|  | Yes, both the DHPC and the EM | 19 (19.6%) | 35 (62.5%) | 40 (67.8%) | 15 (34.9%) |
|  | Only the DHPC | 43 (44.3%) | 16 (28.6%) | 14 (23.7%) | 22 (51.2%) |
|  | Only the EM | 2 (2.1%) | 0 (0.0%) | 0 (0.0%) | 0 (0.0%) |
|  | Neither of them | 33 (34.0%) | 5 (8.9%) | 5 (8.5%) | 6 (14.0%) |
| **Germany** |  | **(N=82)** | **(N=56)** | **(N=55)** | **(N=61)** |
|  | Yes, both the DHPC and the EM | 21 (25.6%) | 30 (53.6%) | 19 (34.5%) | 13 (21.3%) |
|  | Only the DHPC | 27 (32.9%) | 14 (25.0%) | 24 (43.6%) | 21 (34.4%) |
|  | Only the EM | 1 (1.2%) | 0 (0.0%) | 0 (0.0%) | 0 (0.0%) |
|  | Neither of them | 33 (40.2%) | 12 (21.4%) | 12 (21.8%) | 27 (44.3%) |
| **Spain** |  | **(N=83)** | **(N=41)** | **(N=41)** | **(N=79)** |
|  | Yes, both the DHPC and the EM | 15 (18.1%) | 14 (34.1%) | 12 (29.3%) | 14 (17.7%) |
|  | Only the DHPC | 18 (21.7%) | 20 (48.8%) | 13 (31.7%) | 26 (32.9%) |
|  | Only the EM | 1 (1.2%) | 0 (0.0%) | 1 (2.4%) | 2 (2.5%) |
|  | Neither of them | 49 (59.0%) | 7 (17.1%) | 15 (36.6%) | 37 (46.8%) |
| **Sweden** |  | **(N=44)** | **(N=30)** | **(N=31)** | **(N=31)** |
|  | Yes, both the DHPC and the EM | 9 (20.5%) | 7 (23.3%) | 11 (35.5%) | 3 (9.7%) |
|  | Only the DHPC | 9 (20.5%) | 16 (53.3%) | 9 (29.0%) | 7 (22.6%) |
|  | Only the EM | 0 (0.0%) | 0 (0.0%) | 0 (0.0%) | 2 (6.5%) |
|  | Neither of them | 26 (59.1%) | 7 (23.3%) | 11 (35.5%) | 19 (61.3%) |
| **UK** |  | **(N=90)** | **(N=45)** | **(N=47)** | **(N=82)** |
|  | Yes, both the DHPC and the EM | 24 (26.7%) | 18 (40.0%) | 14 (29.8%) | 17 (20.7%) |
|  | Only the DHPC | 18 (20.0%) | 8 (17.8%) | 8 (17.0%) | 10 (12.2%) |
|  | Only the EM | 6 (6.7%) | 2 (4.4%) | 2 (4.3%) | 4 (4.9%) |
|  | Neither of them | 42 (46.7%) | 17 (37.8%) | 23 (48.9%) | 51 (62.2%) |
| **Overall - Weighted results** |  | **(N=671)** | **(N=60)** | **(N=124)** | **(N=298)** |
|  | Yes, both the DHPC and the EM | 147.3 (22.0%) | 30.4 (50.7%) | 54.5 (43.8%) | 64.8 (21.8%) |
|  | Only the DHPC | 210.4 (31.4%) | 17.4 (29.0%) | 39.1 (31.4%) | 103.1 (34.6%) |
|  | Only the EM | 17.7 (2.6%) | 0.2 (0.3%) | 1.1 (0.9%) | 3.0 (1.0%) |
|  | Neither of them | 295.4 (44.0%) | 12.0 (20.0%) | 29.7 (23.9%) | 126.7 (42.6%) |

DHPC: Dear Healthcare Professionals Communication; EM: Educational Materials; GP: General practitioners
